# Disturbances of Modulating Molecules (FOXP3, CTLA-4/CD28/B7, and CD40/CD40L) mRNA Expressions in the Orbital Tissue from Patients with Severe Graves' Ophthalmopathy

**DOI:** 10.1155/2015/340934

**Published:** 2015-01-12

**Authors:** Przemyslaw Pawlowski, Natalia Wawrusiewicz-Kurylonek, Anja Eckstein, Joanna Reszec, Wlodzimierz Luczynski, Kristian Johnson, Adam Kretowski, Alina Bakunowicz-Lazarczyk, Maria Gorska, Jacek Szamatowicz, Lech Chyczewski, Janusz Mysliwiec

**Affiliations:** ^1^Department of Medical Pathomorphology, Cathedral of Biostructure, Medical University of Białystok, 13 Waszyngtona Street, 15-269 Białystok, Poland; ^2^Department of Pediatric Ophthalmology with Strabismus Treatment Unit, Medical University of Białystok, 17 Waszyngtona Street, 15-274 Białystok, Poland; ^3^Department of Diabetology, Endocrinology and Internal Medicine, Medical University of Białystok, 24A Skłodowskiej-Curie Street, 15-276 Białystok, Poland; ^4^Department of Ophthalmology, Essen University Hospital, University of Duisburg-Essen, Hufeland Straße 55, 45 122 Essen, Germany; ^5^Department of Pediatrics, Endocrinology and Diabetology with Cardiology Division, Medical University of Bialystok, 17 Waszyngtona Street, 15-274 Białystok, Poland; ^6^Bayer HealthCare, 10 Kaiser-Wilhelm-Allee, 51373 Leverkusen, Germany; ^7^Department of Gynaecology and Oncologic Gynaecology, Medical University of Bialystok, 24A Skłodowskiej-Curie Street, 15-276 Białystok, Poland; ^8^Department of Nuclear Medicine, Medical University of Bialystok, 24A Skłodowskiej-Curie Street, 15-276 Białystok, Poland

## Abstract

*Purpose.* To evaluate the relationship between the expression of orbital tissue mRNA for FOXP3, CTLA-4/CD28/CD80/CD86, and CD40/CD40 and the severity of Graves' orbitopathy (GO). *Material and Methods.* Orbital tissue was obtained from 26 patients with GO, with mild (*n* = 6) or severe GO (*n* = 20), and 7 healthy controls. The expression of mRNA of FOXP3, CTLA-4/CD28/CD80/CD86, CD40/CD40L was measured by RT-PCR. TCR and CD3 were evaluated by immunohistochemistry. *Results*. Higher mRNA for FoxP3 (relative expression: 1.4) and CD40 (1.27) and lower expression of CTLA-4 (0.61) were found in the GO tissues versus controls. In severe GO as compared to mild GO higher mRNA expression for FoxP3 (1.35) and CD40 (1.4) and lower expression for CTLA-4 (0.78), CD28 (0.62), and CD40L (0.56) were found. A positive correlation was found between FOXP3 mRNA and CD3 infiltration (*R* = 0.796, *P* = 0.0000001). *Conclusions*. The enhanced FOXP3 mRNA expression in GO samples may suggest the dysfunction of FOXP3 cells in the severe GO. The diminished mRNA expression of CTLA-4 in severe GO may indicate inadequate T regulatory function. The enhanced mRNA expression of CD40 in severe GO and negative correlation to CRP mRNA may suggest their role in the active and inactive GO.

## 1. Introduction

Graves' orbitopathy (GO) is an organ-specific autoimmune disease with a multifactorial etiology, involving genes and environmental triggers, with smoking as a first example, that cause autoimmune dysregulation [1–3]. It is unknown why only a small subset of patients with Graves' disease (GD) develops GO. Vulnerability to GO manifestations most likely is connected with the highly specialized function of the orbital tissue, a unique fat depot that cushions the globe [4].

Most orbital disorders are inflammatory [1] suggesting that orbital fat may be especially prone to robust inflammatory reactions. Indeed, as compared with fibroblasts from other sites, orbital fibroblasts show exaggerated inflammatory responses to various stimuli [5, 6]. Orbital fibroblasts express CD40, a costimulatory protein present on the surface of many types of cells, including macrophages, lymphocytes, and thyrocytes. CD4+ T cells expressing the CD40 ligand (CD154) directly activate orbital fibroblasts to proliferate through the formation of CD40-CD154 bridges [7–9].

As with other autoimmune diseases, the initiating event results in loss of tolerance and autoreactivity in the orbit and subsequently leads to mononuclear cell infiltration [10]. Since T cells infiltrating the retrobulbar tissues are likely to play a key role in the pathogenesis of orbital inflammation Forkhead box P3 (FOXP3), CTLA-4/CD28 and its ligands CD80/CD86 have been considered as candidate genes for GO [11, 12]. FoxP3 is a crucial regulatory factor for the development and function of regulatory T cells (T_regs_), and deficiency of the FOXP3 suppresses the regulatory function of T_regs_ [13]. Cytotoxic T lymphocyte antigen 4 (CTLA-4), a molecule specifically and constitutively expressed on T_regs_, plays a key role as a negative regulator of T-cell activation [14]. The CTLA-4 gene was reported to be associated with susceptibility to GO and the G allele at exon 1 CTLA-4(49) A/G polymorphism was correlated with severity of GO [15, 16]. In addition, FOXP3 and CD40 genetic association was evaluated both in human and in animal models of GO [17, 18].

CD80/CD86 (B7-1, B7-2) counter-receptors for CD28/CTLA-4, expressed mainly on the antigen-presenting cells (APC), renders, that activated T cells expressing B7 family molecules could act as APC [12, 19]. Recently, the polymorphisms of the CD86 gene were suggested as genetic markers, which could be helpful for making the diagnosis and prognosis of GO [12].

The present study was designed to investigate the expression of mRNA of the immune regulatory molecules FOXP3, CTLA-4/CD28/CD80/CD86, and CD40/CD40L in the orbital tissues from patients who underwent orbital decompression due to Graves' orbitopathy to assess their role in the inflammatory process.

## 2. Materials and Methods

### 2.1. Patients and Controls

Human orbital fat/connective tissue was derived from the orbital tissue bank at the Department of Ophthalmology and University of Essen. It was obtained from 26 patients with GO (25 females and 1 male) who underwent orbital decompression. The mean age of patients at the time of surgery was 44.5 years (range: 36–49). Normal orbital connective tissues were derived from 7 individuals (6 females and 1 male) undergoing blepharoplastic surgery with no history of GO or any orbital inflammatory disease. The clinical activity score (CAS) of GO was estimated according to Mourits (Mourits et al., 1997). The severity of the eye disease was estimated using a modified classification of no signs or symptoms; only signs, no symptoms; signs only; proptosis; eye muscle involvement; corneal involvement; and sight visual acuity reduction (NOSPECS) described by Eckstein et al. [20]. The mean CAS was 5.92 (1–10) and the mean NOSPECS was 7.46 (2–13).

The study was approved by the Medical Ethics Committee of the University of Essen, Germany and all study participants gave written informed consent.

#### 2.1.1. Grouping of Patients

The patients were grouped according to the CAS and NOSPECS scores on the follow-up at 11–14 months after onset of GO into those with a mild or severe course of GO. The mild signs of GO were described as CAS < 4 (almost inactive GO) and NOSPECS < 5 (mild GO), whilst the severe signs were established as CAS ≥ 4 (still active disease) and/or NOSPECS ≥ 5—“severe GO.” The examiner who classified the patients was blind to the thyroid status and TRAb values.

Clinical characteristics of patients are shown in Table 1.

### 2.2. RNA Extraction and cDNA Synthesis

Total RNA from the tissue was isolated and purified with the use of RNeasy Mini Kit (Qiagen, Valencia, CA, USA), following the manufacturer's protocol. One microgram of total RNA was used to prepare cDNA, and cDNA synthesis was performed using SuperScript First-Strand Synthesis System for RT-PCR (Invitrogen, Carlsbad, CA, USA) according to the manufacturer's instructions in the MJ Research Thermal Cycler (Model PTC-200, Watertown, MA, USA).

### 2.3. RT-PCR and Data Analysis

The levels of transcripts were measured by real-time PCR using human genes QuantiTect Assays and QuantiTect Hs_GAPDH Assay (Qiagen, Valencia, CA, USA) as a normalizer. The following genes were assessed: FOXP3, CD152 (CTLA-4), CD28, CD80 (B7-1), CD86 (B7-2), CD40, CD154 (CD40L), and CRP (C-reactive protein). Real-time PCR was performed in duplicate in 20 *μ*L, using the QuantiTect SYBR Green PCR Master Mix (Qiagen) applying the manufacturer's instructions, and conducted in the Chromo4 Real-Time PCR Detector (BIO-RAD, USA). A standard curve construction was generated by a series of four dilutions of cDNA of the control group sample in reaction to the house-keeping gene, GAPDH. On the basis of these curves, the levels of total chosen gene transcripts were calculated after their normalization to GAPDH. The value of CT was determined by the first cycle number at which fluorescence was higher than the set threshold value.

### 2.4. Evaluation of TCR and CD3 Expression by Immunohistochemical Methods

Following the deparaffinization and rehydration, epitope retrieval was carried out in the EnVision Flex Target Retrieval Solution (DAKO) in high pH. Endogenous peroxidases were blocked by incubating the sections in methanol and 3% hydrogen peroxidase for 20 minutes. Next slides were incubated with mouse monoclonal antibody against TCR receptor (Novocasta) TCR alpha/beta Antibody (R73) in 1 : 100 dilution for 30 minutes in room temperature. Against CD3 receptor, were used rabbit polyclonal antibody (Anti-CD3 antibody (ab5690)) in 1 : 75 dilution for 30 minutes in room temperature. Visualization reagent EnVision (DAKO) was applied for 30 minutes followed by DAB solution for 10 minutes. The slides were then counterstained with hematoxylin and examined under the light microscope. The intensity of immunostaining was evaluated in random 10 fields under 20x magnification. The results were expressed as the percentage of cells with a strong positive staining as follows: ≤10% positive cells: negative (−), between 11% and 50% (+), and >50% positive cells (++) (see Table 2).

## 3. Statistical Analysis

In order to calculate mRNA expression, a comparative CT method was used for relative quantification, that is, 2^−ΔΔCT^, following Livak and Schmittgen [21]. The results were analyzed in Statistica 9.0 for Windows (StatSoft, Poland). Owing to asymmetric data distribution, nonparametric tests were used. Significance levels were calculated in accordance with Mann-Whitney *U* test (differences between the control and examined group). The correlations between the clinical parameters and RT-PCR results were assessed with Spearman's rank correlation test. The level of *P* < 0.05 was regarded as significant. Due to the small amount of samples, the relative values (a comparison between the control and examined samples) below 0.75 were regarded as a decreased gene expression, between 0.75 and 1 as a comparable expression, and above 1 as an increased expression [22]. The data are presented as the relative expression of genes in examined samples compared to control samples with the example of individual data in the case of FoxP3 gene. The graphs were prepared in GraphPad Prism 5.0.

## 4. Results

### 4.1. CD3 and TCR Expression in Lymphoid Cells In Situ Immunohistochemical Evaluation

#### 4.1.1. TCR Expression in Mild GO and Severe GO

**Figure 1:**
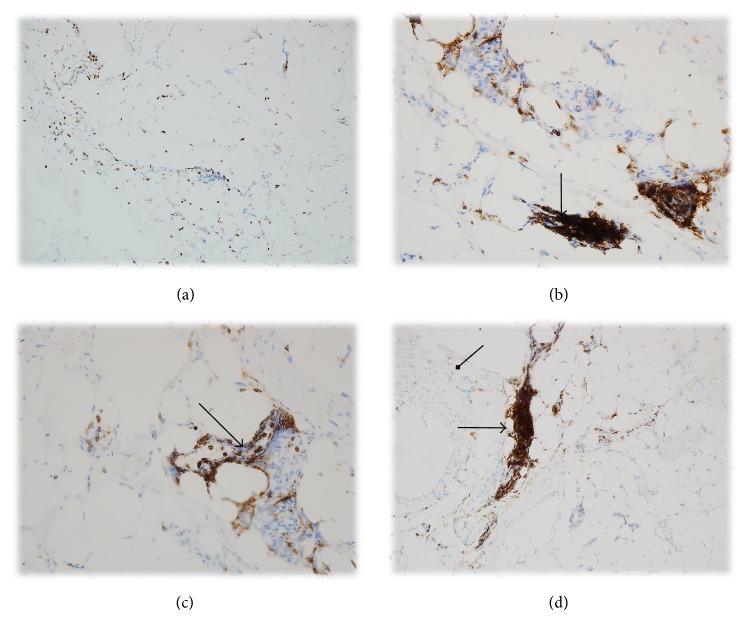
(a) Strong and diffuse TCR expression in lymphoid cells within the mild Graves ophthalmopathy. Magnification: 100x. (b) Focuses of the lymphoid cells aggregation (TCR expression) in the mild Graves ophthalmopathy. Magnification: 200x. ((c), (d)) Strong and diffuse TCR receptor expression in lymphocytes within severe Graves ophthalmopathy. Magnification: 200x, 100x (the arrows show T cells and fibroblasts).

Focal lymphoid cells aggregation with TCR expression in the mild Graves ophthalmopathy was seen in (4/6 samples) 66.66 % with mild GO and similarly in 65% with severe GO (13/20 samples). However, in 7/10 (35%) with severe GO, the expression of TCR in the lymphoid cell was evaluated as (++) (Figures 1 and 5).

In addition, no expression of CD3 or TCR was revealed in the control tissue specimens (Table 3).

#### 4.1.2. CD3 Expression in Mild GO and Severe GO

**Figure 2:**
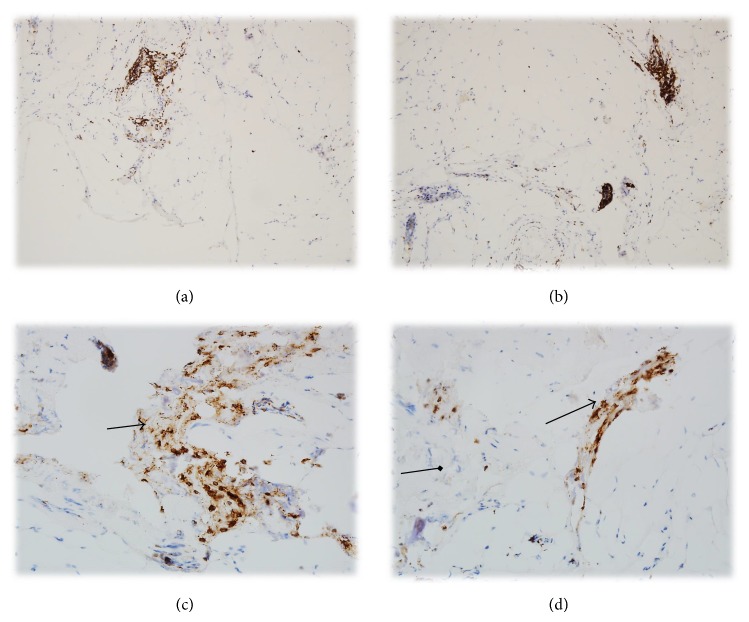
((a), (b)) CD3 expression in T cells within the mild Graves ophthalmopathy. Magnification: 100x. ((c), (d)) CD3 strong expression in T cells within the severe Graves ophthalmopathy. Magnification: 200x (the arrows show T cells and fibroblasts).

**Figure 3:**
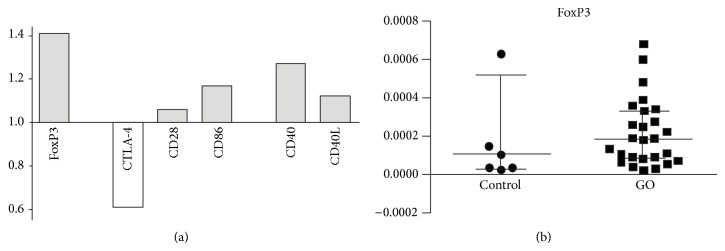
(a) Quantitative RT-PCR validation of the mRNA expression changes for the CD86/CD28/CTLA-4: FOXP3 and CD40/CD40L molecules in GO (mild + severe) orbital tissues compared to control tissue samples. The values above “1.0” on the *y*-axis show the relative higher expression in GO versus control samples and values below “1.0” indicate relatively lower expression. (b) The individual data from RT-PCR displaying the expression of mRNA for transcription factor FoxP3 in control and examined (GO) samples (medians and 25th–75th percentiles) (*P* < 0.05).

**Figure 4:**
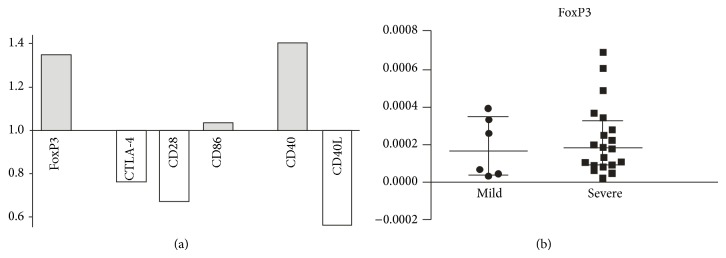
(a) Quantitative RT-PCR validation of the mRNA expression changes for assessed molecules in the severe GO compared to mild GO samples. The values above “1.0” on the *y*-axis show the relatively higher expression in the severe versus mild GO samples and values below “1.0” indicate relatively lower expression. (b) The individual data from RT-PCR displaying the expression of mRNA for transcription factor FoxP3 in severe GO and mild GO samples (medians and 25th–75th percentiles).

**Figure 5:**
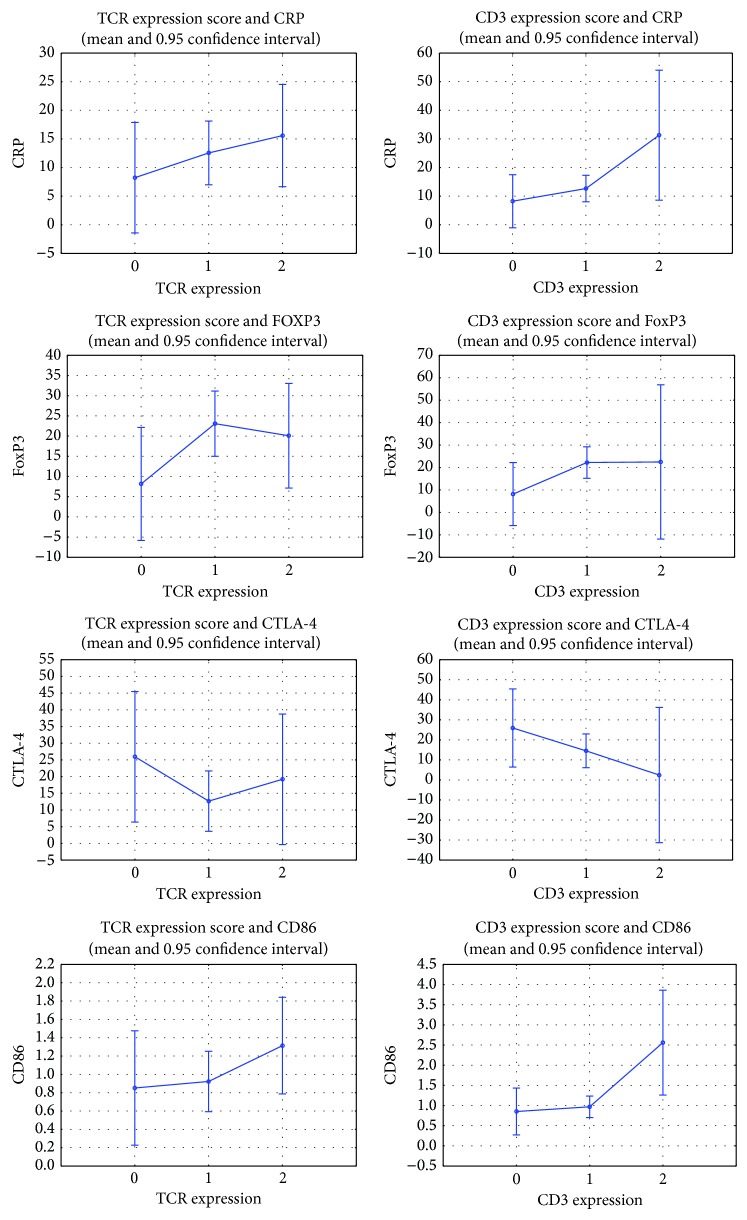
TCR and CD3 immunohistochemical grading score groups' (0: ≤10% positive cells; 1: 11%–50%, 2: >50% cells) mean and confidence values of CRP, FOXP3, CTLA-4, and CD86 mRNA.

66.66% with mild GO (4/6) and 95% with severe GO (19/20) had focal CD3 lymphoid cells infiltrations in the orbital tissue. In addition, in one patient with severe GO (5%), the infiltration was strong and diffused (Figures 1, 2, 3, 4, and 5 and Table 3)

### 4.2. The mRNA Expression in GO Tissues and Control Samples

The mRNA for FoxP3, CRP, CD40, and CD86 were present in all the samples (*N* = 33, 100.0%). The mRNA for CTLA-4 was detected in 3/7, that is, 42.8% of controls, and 19/26, that is, 73.0% of GO tissue samples. The mRNA for CD28 molecule was noted in 2/7, that is, 28.5% of controls, and 10/26, that is, 38.4% of GO tissue samples. The mRNA for CD40L molecule was present only in 1/7, that is, 14.2% of controls, and 8/26, that is, 30.7% of GO tissue samples. The mRNA for CD80 was not detected in controls (0/7) and only in 8/26, that is, 30.7%, of GO tissue samples. No statistically significant differences were noted between control and examined samples concerning the expression of assessed genes. However, according to our further analysis based on quantitative RT-PCR validation of the mRNA expression changes (as stated in statistical analysis), higher amounts of mRNA for FoxP3 (relative expression: 1.4), CD28 (1.06), CD40 (1.27), CD40L (1.17), and CD86 (1.17) and lower expression of CTLA-4 (0.61) and CRP (0.62) were found in GO as compared to control tissues (see Figure 3).

Further, the expression of mRNA between tissues from patients with severe to mild GO was compared. No significant differences were noted in accordance with Mann-Whitney *U* test. However, in the analysis of relative amounts of mRNA, we found higher expression of mRNA for FoxP3 (relative expression: 1.35) and CD40 (1.4) and lower expression of mRNA for CTLA-4 (0.78), CD28 (0.62), and CD40L (0.56) in severe GO as compared to mild GO tissue samples. The relative expression of mRNA for assessed molecules is presented in Figure 4.

We found no correlation between the clinical data and results from RT-PCR. However, CRP mRNA correlated positively with FOXP3 (*R* = 0.520 and *P* = 0.03) and negatively with CD40 values (*R* = −0.757 and *P* = 0.000001). In addition, a positive correlation was found between FOXP3 mRNA and CD3 infiltration (*R* = 0.358, *P* = 0.05) and a strong correlation was found between TCR and CD3 expression (*R* = 0.796, *P* = 0.0000001) (see Table 4).

## 5. Discussion

The explanation why the connective tissue of the orbit and skin should be singled out for activation in GD concerns possible intrinsic differences in the residential orbital and leg cells as setting up these regions for disease involvement in GD [4]. Among other immunoregulatory molecules, CTLA-4 and CD40 in GD and GO have been evaluated most extensively [15, 23]. In an early study, Vaidya et al. demonstrated that the G-carrying genotypes of the* CTLA4A/G* polymorphism are associated with an increase in risk of GO and are independent of male sex, smoking, and previous radioiodine administration [24]. Since then, many studies have evaluated the CTLA-4 polymorphism in different ethnic groups and its association with ophthalmopathy and Graves' disease [25]. For CTLA-4 function SNP C-318 T, SNP A49G (alanine/threonine polymorphism) and microsatellite- sequence in 3′UTR was determined [26]. Most recently, Borodic et al. described a case report on treatment with ipilimumab, the monoclonal antibody blocking CTLA-4 mediated T cell suppression, that has led to GO-like syndrome [27]. The decreased expression of the CTLA-4 mRNA and increased expression of its counter-receptor B7-2 (CD86) in the orbital tissue from GO may explain that patients with GD develop severe GO due to the increased autoimmune reactivity in the orbital venue. Saverino et al. found elevated soluble form of CTLA-4 (sCTLA-4) in serum from patients with autoimmune thyroid disease suggesting a possible role in immune response dysregulation [28]. Thus, the systemic elevation may be caused by increased cleavage of the surface CTLA-4 and lack of its expression in inflammation site in the orbit.

The increased CD86 mRNA expression found in GO orbital samples may suggest that CD86 costimulation promotes a direct immune response toward Th2 lymphocytes development in the orbital tissue [29]. Th2 differentiation has been shown to be involved in the humoral immune response involving B cells and antibodies production. Sigal et al. found that the CD86 costimulation was more critical for cytotoxic T lymphocytes generation [30].

Similar to CD86, CD40 and CD40L were identified as important B cell activation factors [31]. Most recently, both CD40 and CD86 were found to be elevated in the thyroid of the animal model of GD [32]. In agreement with that, we found an increased level of CD40 mRNA in the orbital tissue samples. In addition, a single nucleotide polymorphism (SNP) C/T in the untranslated region of the CD40 gene has been associated with susceptibility to Graves' disease in Caucasian [33] and Korean populations [2]. This SNP may alter the translational efficiency of CD40 protein [34]. Jacobson et al. showed that the T-allele makes 15.5% less CD40 than the C-allele, demonstrating that the effect of the single-nucleotide polymorphism (SNP) on CD40 expression is at the level of translation [34]. Human B cells harboring this SNP expressed 39% and 27% higher levels of surface CD40 at rest and activation, respectively, compared with controls. Moreover, this SNP has been linked with the increased production of autoantibodies [35]. However, no difference was detected in steady state CD40 mRNA expression in GD [34].

It is possible that the elevated levels of surface CD40 might amplify cell activation during tissue injury, potentially predisposing the host to GD and perhaps to GO [36]. Since ligation of CD40L on T cells by CD40 B cells or other APC was shown to be necessary for efficient activation of T cell effector functions, elevated serum concentrations of serum CD40 and CD154 could suggest the systemic activation and explain the increased CD40/CD40L mRNA level in the orbital tissue as a pathway leading to inflammatory infiltration in GO [37].

Direct evidence that any particular candidate antigen or orbital cell type is an autoimmune target in GO has been difficult to obtain due to the lack of available human orbital tissue. However, the loss of tolerance could anyway be the trigger of GO. In agreement with that, we found that the level of FOXP3 mRNA was elevated in GO patients comparing to controls. In addition, the expression of FOXP3 mRNA was also higher in patients with the severe course of GO than in patients with the mild course of GO. These disturbances could be elucidated by the fact that, by preventing the activation of autoreactive pathogenic cells, CD4^+^CD25^+^FOXP3^+^ regulatory T lymphocytes (T_regs_) have a critical role in the maintenance of self-tolerance and thus in the prevention of autoimmune disease [13]. Hence, the autoimmune response might be more enhanced in GO especially in patients with severe GO. It has been shown that the incubation with polyclonal rabbit anti-T lymphocyte (rATG) globulin increased the frequency of PBMCs of GO patients expressing T_regs_-markers (CD25, FOXP3). Kahaly et al. have found that FOXP3/CD4 rATG-induced T_regs_ marker was more intensively expressed on GO peripheral blood leucocytes (PBLs) than on GD or normal PBLs [38]. Interestingly, FOXP3 mRNA from peripheral blood was equal in patients with active GD to that in controls [39]. It is possible that in our study the enhanced FOXP3 mRNA could be linked with a polymorphism or a dysfunction of FOXP3 that attempted to compensate the immunological response in its overexpression [18].

## 6. Conclusions 

The enhanced FOXP3 mRNA expression in GO samples and its correlation with CD3 and CRP may suggest the involvement and perhaps dysfunction of FOXP3 lymphoid cells in the pathogenesis of severe GO. The diminished mRNA expression of CTLA-4 in severe GO may indicate inadequate T regulatory function of this molecule in severe course of GO. The enhanced mRNA expression of CD40 in severe versus mild GO and negative correlation to CRP mRNA may suggest their role not only in active but also in the late inactive phase of GO.

## Figures and Tables

**Table 1:** Characteristics of patients.

	Severe course of GO	Mild course of GO	Mild + severe GO
Number of patients	20	6	26
Age	42.3 (36–46)	46.2 (42–49)	44.5 years (36–49)
Gender	19F; 1M	6F	25F; 1M
Smokers	88%	50%	77%
TRAb (mean ± SD)	23 ± 11.57	2.56 ± 1.3	18.89 ± 13.59
CAS	7.1 (4–10)	2 (1–3)	5.92 (1–10)
NOSPECS	8.55 (5–13)	3.83 (2–5)	7.46 (2–13)
Proptosis right	18 (14.5–25)	16.25 (14–18)	17.5 (14–25)
Proptosis left	19.5 (15–25)	16.5 (14–21.5)	17.5 (14–25)

**Table 2:** Immunohistochemical evaluation grading score for TCR and CD3 of each specimen.

Number	Group	TCR	CD3
1	Control	0	0
2	Control	0	0
3	Control	0	0
4	Control	0	0
5	Control	0	0
6	Control	0	0
7	Control	0	0
8	Mild GO	0	0
9	Mild GO	1	1
10	Mild GO	1	1
11	Mild GO	1	1
12	Mild GO	1	1
13	Mild GO	0	0
14	Severe GO	1	1
15	Severe GO	1	1
16	Severe GO	1	1
17	Severe GO	1	1
18	Severe GO	2	1
19	Severe GO	1	1
20	Severe GO	1	1
21	Severe GO	2	1
22	Severe GO	2	1
23	Severe GO	2	2
24	Severe GO	1	1
25	Severe GO	2	1
26	Severe GO	2	1
27	Severe GO	1	1
28	Severe GO	1	1
29	Severe GO	1	1
30	Severe GO	1	1
31	Severe GO	1	1
32	Severe GO	2	1
33	Severe GO	1	1

Legend for Table 2: Immunohistochemistry scores are as follows:

0: less than 10% positive in 10 representative high power fields (HPF),

1: 11%–50% positive cells in 10 HPF,

2: more than 50% positive cells in 10 HPF.

Immunohistochemistry was done using DAB chromogen (brown staining).

**(a):** 

TCR expression	Control	Mild GO	Severe GO	Sum
≤10%	7	2	0	9
[%]	100.0%	33.34%	0.00%	
[10%–50%]	0	4	13	17
[%]	0.00%	66.66%	65.00%	
>50%	0	0	7	7
[%]	0.00%	0.00%	35.00%	

**(b):** 

CD3 expression	Control	Mild GO	Severe GO	Sum
≤10%	7	2	0	9
[%]	100.0%	33.34%	0%	
[10%–50%]	0	4	19	24
[%]	0.0%	66.66%	95%	
>50%	0	0	1	1
[%]	0.00%	0.00%	5%	

**Table 4:** Spearman's correlation table with number of samples, *R*, and *P* value.

	*N*	*R*	*P* value
TCR expression & CD3 expression	32	0.796	**0.0000001**
CD3 expression & CTLA-4	20	−0.375	0.1
CD3 expression & Fox	**32**	**0.358**	**0.05**
CD3 expression & CD40	32	−0.124	0.5
CD3 expression & CD86	32	0.186	0.3
CRP & Fox	**32**	**0.520**	**0.003**
CRP & CD40	**32**	**−0.757**	**0.000001**
